# Corner coil heating mode improves the matrix uniformity of cooked rice in an induction heating cooker

**DOI:** 10.3389/fnut.2022.1038708

**Published:** 2022-11-15

**Authors:** Jinxi Kong, Jinxuan Tao, Shanlin Fu, Ya Wen, Siming Zhao, Binjia Zhang

**Affiliations:** ^1^Key Laboratory of Environment Correlative Dietology (Ministry of Education), College of Food Science and Technology, Huazhong Agricultural University, Wuhan, China; ^2^Department of Home Appliance Technology Research, Zhuhai Gree Electric Appliances Co., Ltd., Zhuhai, China; ^3^Chongqing Key Laboratory of Speciality Food Co-Built by Sichuan and Chongqing, College of Food Science, Southwest University, Chongqing, China

**Keywords:** rice, induction heating cooker, heating mode, texture uniformity, digestion

## Abstract

Nowadays, an increasing number of people worldwide use induction heating cookers to cook rice for consumption. This work reveals the influence of induction heating cooker heating modes on the quality attributes of cooked rice. Three heating modes, including bottom coil heating mode (mode 1), corner coil heating mode (mode 2), and side coil heating (mode 3), were used. Among the three modes, mode 2 allowed for an intermediate heating rate during rice cooking. For mode 2, the minimized temperature difference between the upper layer (including the central upper layer and peripheral upper layer) and the lower layer (including the central lower layer and peripheral lower layer) can reduce the effect of water absorption time difference on rice quality. Consequently, the rice cooked using mode 2 exhibited improved matrix uniformity, as indicated by the similar moisture content (59.92–61.89%), hardness (15.87–18.24 N), and water mobility (the relaxation time and peak area of the third relaxation peak) of rice samples at four different positions in the pot. The rice cooked by mode 2 showed better texture appearance and a more uniform porous microstructure. Consistently, the cooked rice samples by mode 2 at different positions did not show substantial differences in their starch digestion features.

## Introduction

Rice is one of the most important staple foods for humans, feeding more than half of the world’s population. During cooking, rice kernels undergo many changes, such as hydration, swelling (1), cracking (2, 3), starch gelatinization, and leaching of solids (4–6), all of which may contribute to the final texture of cooked rice.

In recent years, researches have studied various cooking methods to maximize the taste and edible value of cooked rice. Many studies have shown that both cooking temperature and cooking time have significant impacts on the physicochemical properties (7–10). Additionally, high hydrostatic pressure soaking could increase the hydration degree of rice, leading to a smaller network of channels (11). It has also been indicated that changing the cooking mode can slow the digestion of starch in brown rice (12). Researchers have attempted to correlate the physicochemical properties of cooked rice with different cooking processing parameters. A recent study demonstrated heating rates influenced the final texture of rice by affecting the water distribution and starch gelatinization of rice during cooking (13). Furthermore, with a gradual decrease in the heating power of cooked rice during cooking, the appearance, taste, comprehensive score, and sensory score of cooked rice are enhanced (14).

Due to convenience, induction heating cookers have become the main cooking appliances in family cooking and its primary mechanism involves electromagnetism and heat transfer domains (15). However, most studies investigated the characteristics of a small amount of rice, or only some of the rice from a larger pot used to feed a whole family (16–19). When the amount of rice cooked in the rice cooker increases, differences in the water absorption and temperature at different positions in the pot can be observed, and these differences lead to variability in its texture (13, 20). Research on the eating quality differences in rice prepared in a rice cooker is scarce. Moreover, the electromagnetic coil, as the heating source, is an important factor in the formation of a temperature field in the pot. Studies on the influence of heat source position on rice quality have not yet been reported.

Therefore, in this study, rice quality using three typical heat sources in induction heating cookers by changing the positions of the electromagnetic coil were firstly investigated. The temperature distribution characteristics, the differences in appearance, moisture, texture, and digestion characteristics of cooked rice were examined, which is helpful to guide the design of the electromagnetic coil of induction heating cookers.

## Materials and methods

### Experiment materials

The indica rice used in this study was purchased from local supermarkets in Zhuhai City, with a rice moisture content of 12.2% and dry base starch level of 68.3% (dry basis). Two enzymes α-amylase (activity: 50 U/mg) and amyloglucosidase (activity: 100 U/mg) were purchased from Shanghai Yuanye Biological Co., Ltd. (Shanghai, China). A glucose oxidase/peroxidase kit (GOPOD reagent) was provided by Shanghai Rongsheng Bio-pharmaceutical Co., Ltd. (Shanghai, China). All chemical reagents were of analytical grade.

### Rice cooking temperature acquisition

**FIGURE 1:**
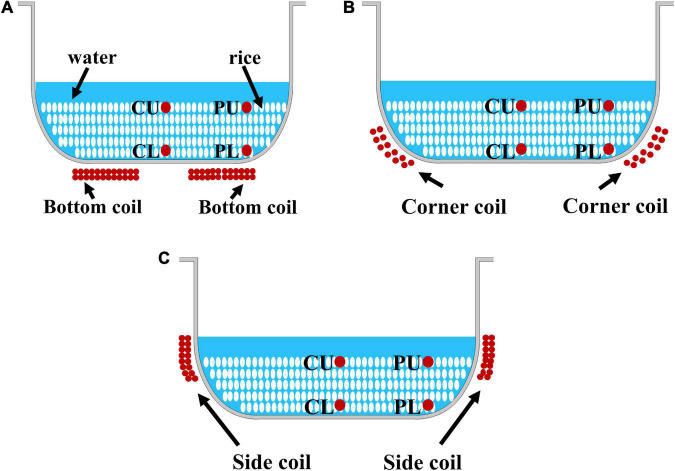
Schematics of different heating modes and sampling points. **(A)** Mode 1. **(B)** Mode 2. **(C)** Mode 3. CU, central upper layer; PU, peripheral upper layer; CL, central lower layer; and PL, peripheral lower layer.

Six hundred grams of rice was quickly washed with water three times, combined with pure water at a rice:water mass ratio of 1:1.4, and cooked in an electromagnetic heating rice cooker (Gree Electric Appliances Co., Ltd., Zhuhai, China) to prepare a rice sample. During the cooking process, the heating power and heating time were the same in the three heating modes, and the electromagnetic coils at three typical positions in the rice cooker were used separately for heating. The bottom coil was used in mode 1 (Figure 1A); the corner coil was used in mode 2 (Figure 1B); and the side coil was used in mode 3 (Figure 1C). The cooking temperature was acquired according to a previously reported method, with slight modification (21). Prior to cooking, T-type thermocouples were placed at four different positions in the central upper layer (CU), peripheral upper layer (PU), central lower layer (CL), and peripheral lower layer (PL) in the inner pot (Figure 1) due to its symmetrical structure, and a temperature acquisition instrument (34970A, Keysight Technologies Co., Ltd., Guangzhou, China) was used for real-time recording of the temperature field data in the pot under different heating modes.

### Observation of rice appearance

In order to investigate the difference in rice morphology under different modes, the appearance of cooked rice was observed using a high-resolution stereomicroscope (VHX-7000, KEYENCE, Osaka, Japan) with a magnification of 20× (22).

### Rice low-field NMR test

The moisture characteristics of rice under different heating modes and with different heat sources were measured on a LF-NMR spectrometer (Niumai Instruments, NMI20-015V-I, Suzhou, China), as previously described (23, 24). In order to ensure the parallelism of the experiment and the reliability of the results, each measurement was conducted at the same fixed time period. The preparation and measurement of the rice samples were completed within 40 min of cooking, so as to prevent the samples from being exposed to the air for too long and losing water, which would affect the experimental results. The measurement parameters were as follows: P1 = 13.52 μs, TW = 4000 ms, and P2 = 26.00 μs. For each sample, six parallel tests were conducted. After the measurement, the data were saved, and the inversion software (NMR relaxation time inversion fitting software Ver4.09 provided by Shanghai Niumai Electronic Technology Co., Ltd. Shanghai, China) was used to obtain the distribution of T_2_.

### Scanning electron microscope observation of rice cross-section

The cooked rice samples were immediately dried by a vacuum freeze dryer (ALPHA1-4, Marin Christ Inc., Osterode, Germany), following a previously reported method (25). After drying, the rice sample was broken with tweezers to observe its cross-section. The samples were adhered with double-sided tape with the cross-section facing upward and gold-plated under vacuum. The cross-section structure of the sample was observed using a scanning electron microscope at 2000 × magnification (TESCAN CLAR LMH, TESCAN Trading Co., Ltd., Brno, Czechia).

### Determination of physicochemical properties of rice

After cooking was finished, the rice was quickly sampled from the different positions, as shown in Figure 1, to measure the moisture content, expansion ratio (ER), and texture properties of the rice. The moisture content of the rice sample was determined using the standard association of official analytical chemists (AOAC) method (26). The expansion rate was determined *via* the drainage method with minor modification, as reported by Pan et al. and Wang et al. (27, 28). Briefly, 50 ml of water was transferred to a 100 ml measuring cylinder. Twenty-five grams of rice was added to the water and immediately stirred with a glass rod to evenly disperse the rice grains in the water. The difference in volume was the taken as the volume of rice. The expansion rate of rice from different positions was calculated using the ratio of the volume of rice from different positions to the volume of raw rice. For each rice sample, three parallel experiments were performed.

The texture properties of the rice samples were determined in a two-cycle compression mode (TPA mode) using a texture profile analyzer (TMS-TOUCH, Food Technology Corporation, Virginia, USA), as previously described (25). Three whole grains of rice were placed in parallel on an aluminum plate. During the measurement process, a cylindrical probe with a diameter of 38.1 mm was used for compression, with a deformation amount of 70%. The detection speed was 120 mm/min, and the initial force was 0.5 N. Based on the force-time curves obtained in the test, the firmness and stickiness of the rice were determined. Six parallel experiments were carried out for each rice sample.

### Determination of *in vitro* digestion characteristics of rice starch

As shown in Figure 1, 50 g of rice was taken from each sampling point, combined with 150 ml of anhydrous ethanol, quickly stirred and dispersed with a glass rod, and dehydrated. After suction filtration, the samples were dried at 40°C to constant weight, pulverized with a pulverizer, passed through a 100-mesh sieve, and placed in a desiccator.

Referring to the method reported by Liu et al. (29) with slight modification, the rice powder containing about 90 mg of starch was placed in a 50 ml conical flask. Then, 6 ml of distilled water and 10 ml of sodium acetate buffer solution (pH 6.0) were added, and the mixture was heated for 10 min at 37 °C. Next, 5.0 ml of mixed enzyme buffer solution containing 42 U/ml α-amylase and 42 U/ml amyloglucosidase was added, and the sample solution was hydrolyzed in a water bath at 37 °C for 0, 10, 20, 30, 60, 90, 120, and 180 min, respectively. At 180 min, 0.2 ml of the hydrolysis solution was removed, and 0.8 ml of absolute ethanol was added to inactivate the enzyme. The mixture was centrifuged at 4,000 r/min for 10 min. The absorbance value of the samples was measured using a glucose oxidase/peroxidase kit (GOPOD reagent) with 5.55 mmol/L glucose reagent as the standard. The starch hydrolysis rate of the sample at a certain time was calculated according to the following formula:


(1) $$starchhydrolysisrate\left(\%\right)=\frac{\frac{A_{s\,a\,m\,p\,l\,e}}{A_{s\,t\,a\,n\,d\,a\,r\,d}}\cdot c_{s\,t\,a\,n\,d\,a\,r\,d}\cdot V_{t\,o\,t\,a\,l}\cdot 0.9\cdot f}{m_{s\,t\,a\,r\,c\,h}}\times 100$$

where *A*_*sample*_ is the absorbance of the sample; *A*_*standard*_ is the absorbance of the glucose standard solution; *C*_*standard*_ is the glucose standard solution concentration (5.55 mmol/L); *V*_*total*_ is the total volume of the digestion solution; *f* is the dilution folds; 0.9 is the conversion coefficient from glucose into starch; and *m*_*starch*_ is the product of the mass of the sample and the percentage of contained starch. The contents of rapidly digestible starch (RDS), slowly digestible starch (SDS), and resistant starch (RS) were calculated according to a previously reported method (30). The *in vitro* starch digestion process was consistent with first-order kinetics, and the formula was as follows:


(2) $$C_{t}=C_{\infty }\,\left(1-e^{k\,t}\right)$$

where *C*_*t*_ is the percentage of hydrolyzed starch when the digestion time is *t*; *C*_∞_ is the percentage of hydrolyzed starch in the case of the hydrolysis equilibrium during the digestion process; *k* is the first-order kinetic constant; and *t* is the digestion time. The transfer function of the logarithm of slope (LOS) plot was obtained by taking the logarithm of the first derivative of the first order equation using the following formula:


(3) $$l\,n\,\frac{d\,c}{d\,t}=-k\,t+l\,n\,\left(C_{\infty }\,k\right)$$

where *ln (dc/dt)* is the logarithm of the slope. The parameters *C*_∞_ and *k* were calculated by linear fitting of LOS and starch hydrolysis time *t*, and the starch digestion behavior of different rice powder samples was further evaluated.

### Statistical analysis

The data are expressed as mean values ± standard deviations. Origin (version 2021 for windows, Origin-Lab, Northampton, MA, USA) was used to draw plots. The comparison of means was conducted by SPSS software (version 17.0 for windows, SPSS Inc., Chicago, IL, USA).

## Results and discussion

### Temperature distribution during cooking

**FIGURE 2:**
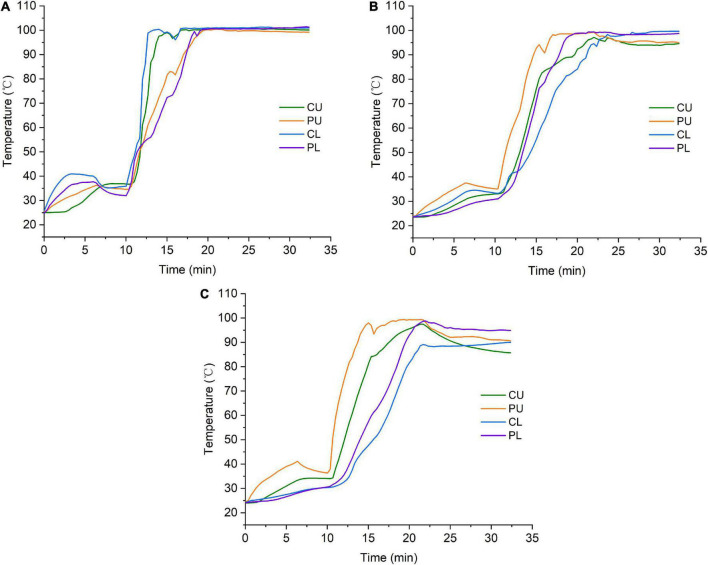
Temperature distribution under different heating modes. **(A)** Mode 1. **(B)** Mode 2. **(C)** Mode 3. CU, central upper layer; PU, peripheral upper layer; CL, central lower layer; and PL, peripheral lower layer.

The temperature distribution curves under three specific heating modes are shown in Figure 2, and significant differences were found between the three cooking modes. The cooking process could be divided into the rice soaking stage (0–10 min), the rapid heating stage (10–20 min), and the high-temperature maintenance stage (20–32 min). Temperature differences were found at the initial rice soaking stage of mode 1, in which the temperature of the central lower layer (CL) was the highest and that of the central upper layer (CU) was the lowest. At the end of the soaking stage, the temperature of the entire pot maintained approximately 30–35°C. During the rapid heating stage, the temperature at the four sampling points increased sharply and reached boiling (100°C) with different heating rates. The heating rate at the CL and the CU was faster than that at the PU and the PL. During the high-temperature maintenance stage, the rice temperature at four different positions (CU, PU, CL, and PL) in the pot was maintained at 100°C until the end of cooking. Under mode 2, the temperature of PU was higher than that of the other three sampling points during the rice soaking and rapid heating stages. Furthermore, the rate of increasing temperature at different sampling points was slower and more consistent under mode 2 than under mode 1. However, the temperature at CU and PU gradually decreased and maintained at 95°C during the high-temperature maintenance stage. For rice heated by mode 3, the temperature of the rice in different layers of the rice cooker was not maintained at 100°C. In particular, the temperature at CU decreased fastest, due to the distance from the heat source and the heat dissipation of the rice cooker. These results indicate differences in temperature distribution between the three cooking modes. Among them, the heating rate of mode 1 was the fastest. The heating rate of the mode 2 was moderate, and the heating temperature of mode 3 was the slowest. Moreover, the temperature difference at various sampling points was the largest for mode 3.

### Morphologic properties of cooked rice

**FIGURE 3:**
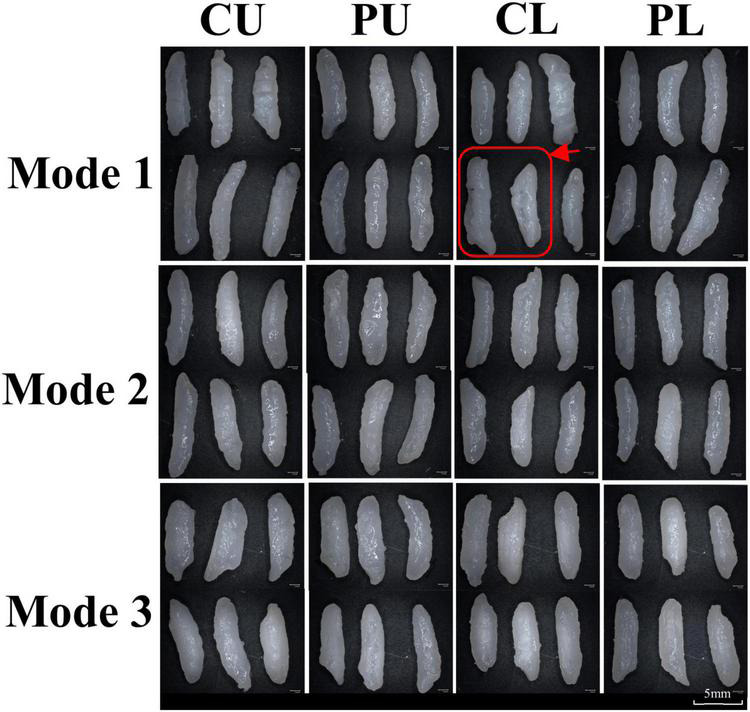
Morphological changes in rice under different heating modes. The red line marked damaged rice. CU, central upper layer; PU, peripheral upper layer; CL, central lower layer; and PL, peripheral lower layer.

The morphology of rice samples under different heating modes is shown in Figure 3. The rice surface cooked by modes 2 and 3 was relatively smooth and intact, for a lower degree of starch gelatinization resulted in higher density of the tissue (31). A slower heating rate during the heating stage and lower maintained temperature (85–95°C) during the high-temperature maintenance stage resulted in insufficient gelatinization of rice sample of mode 3. The cooked rice of mode1-CU and mode1-CL were prone to disruption and deformation (Figure 3), compared with other samples. This is probably due to the higher heating rate and longer maintenance time at a high temperature (above 98°C) of mode1-CU and mode1-CL (27, 32). Furthermore, these changes led to the disruption of the rice surface, due to contact between boiling water and the rice surface (33). Tamura et al. (18) found starch gelatinization between 85 and 100°C damaged the cell wall along the rice surface and changed the rice shape.

### Microstructure of cooked rice

**FIGURE 4:**
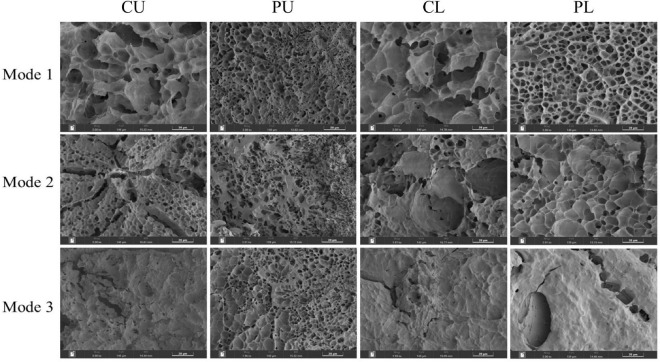
Morphological structure of the center of cooked rice cross-sections under different heating modes.

In order to clarify the morphological and textural properties of cooked rice, Scanning electron microscope (SEM) was employed to observe the microstructure of cooked rice at the center (Figure 4). Significant differences in microstructure between the different heating modes were observed. For mode 2, the cooked rice at different sampling positions showed porous structures with similar pore size. Compared with other samples, rice cooked at CU and CL positions of mode 1 had large cavities. This may be due to the fact that kernels located at CU and CL (mode 1) absorbed more water during cooking. For CU, CL, and PL (mode 3), most of the starch granules were tightly packed because of lower moisture content in the inner areas (31, 34), which was consistent with the results observed by stereomicroscope.

### Moisture characteristics of rice

To gain further insight into water state differences of cooked rice at a molecular lever, the cooked rice was analyzed using proton relaxation measurements. Similar populations were observed for the cooked rice at different cooking modes (Table 1). The range of T_2_ values of the cooked rice was T_21_ (0.1–1 ms), T_22_ (1–10 ms), and T_23_ (10–100 ms), which were named according to the order they appeared in the time scale.

**TABLE 1:** Moisture characteristics of rice under different heating modes (*n* = 6, mean ± STDEV).

Mode	Position	T21 (ms)	A21 (%)	T22 (ms)	A22 (%)	T23 (ms)	A23 (%)
Mode 1	CU	0.46 ± 0.09^a^	3.25 ± 1.48^c^	3.11 ± 0.56^abc^	9.75 ± 1.79^a^	50.41 ± 6.14^a^	86.75 ± 1.48^a^
	PU	0.51 ± 0.33^a^	6.33 ± 4.11^bc^	2.24 ± 0.29^bc^	8.68 ± 6.17^a^	36.25 ± 1.56^c^	84.00 ± 2.16^ab^
	CL	0.56 ± 0.17^a^	9.00 ± 2.94^abc^	3.71 ± 0.36^a^	8.00 ± 0.82^a^	42.28 ± 3.99^b^	83.00 ± 3.56^abc^
	PL	0.40 ± 0.20^a^	7.50 ± 2.69^abc^	2.39 ± 0.95^abc^	10.00 ± 3.08^a^	32.74 ± 0.48^cd^	82.75 ± 1.09^abc^
Mode 2	CU	0.38 ± 0.10^a^	8.50 ± 0.50^abc^	2.19 ± 0.45^bc^	10.50 ± 1.50^a^	35.41 ± 1.23^c^	81.00 ± 1.00^abc^
	PU	0.40 ± 0.10^a^	14.00 ± 4.36^ab^	2.78 ± 0.60^abc^	7.50 ± 3.50^a^	31.10 ± 1.19^cd^	78.75 ± 2.28^bc^
	CL	0.29 ± 0.09^a^	5.50 ± 3.50^bc^	2.36 ± 0.79^abc^	12.50 ± 2.50^a^	33.85 ± 4.09^cd^	82.00 ± 1.00^abc^
	PL	0.21 ± 0.03^a^	6.50 ± 3.50^bc^	2.15 ± 0.58^c^	12.50 ± 1.50^a^	34.21 ± 1.19^cd^	81.50 ± 1.50^abc^
Mode 3	CU	0.39 ± 0.24^a^	7.33 ± 4.99^abc^	3.61 ± 0.20^ab^	9.33 ± 2.05^a^	44.14 ± 2.55^b^	83.00 ± 4.32^abc^
	PU	0.46 ± 0.28^a^	7.25 ± 3.56^abc^	2.64 ± 0.82^abc^	9.75 ± 4.44^a^	35.15 ± 2.15^c^	82.75 ± 1.92^abc^
	CL	0.48 ± 0.28^a^	15.50 ± 0.50^a^	2.12 ± 0.44^c^	7.50 ± 4.50^a^	27.79 ± 0.96^d^	77.00 ± 4.00^c^
	PL	0.34 ± 0.12^a^	9.25 ± 4.82^abc^	2.30 ± 0.37^abc^	11.50 ± 1.80^a^	30.85 ± 1.51^cd^	79.50 ± 3.57^bc^

The values in the table are the mean ± standard deviation and the different lower-case letters in the same column represent significant differences (*p* < 0.05).

As shown in Table 2, the peak area of T_23_ varied from 77–86% for the three cooking modes, presenting typical T_23_ predominant peak characteristics of the cooked rice (35, 36). The time scale and peak area of the relaxation singles were different, indicating varied mobilities of the polymers and water state in cooked rice under different cooking modes (25). In mode 2, there was no significant difference in the relaxation time of T_23_ and peak area of T_23_ between different positions in the pot. In contrast, significant differences between modes 1 and 3 were observed, which were consistent with the moisture content distribution. In mode 1, the rice samples at CU and CL had larger holes than those at PU and PL (Figure 3). This is because more bulk water was found at PU and PL, leading to a higher relaxation time of T_23_ (35, 37). In mode 3, the upper layer had a larger T_23_ peak area than the lower layer, indicating higher mobility of water in the upper layer. With regard to the microstructure of the cooked rice, the insufficient gelatinization of rice resulted in a tightly packed tissue structure with fewer and smaller holes. Thus, fewer water molecules were intercepted (35).

**TABLE 2:** Moisture content, expansion ratio and texture properties of rice under different heating modes.

Mode	Position	Moisture content (%wb)	ER (%)	Hardness (N)	Adhesiveness (mJ)
Mode 1	CU	68.03 ± 0.26^b^	245.5 ± 5.63^b^	11.04 ± 1.02^bc^	0.35 ± 0.19^a^
	PU	59.21 ± 1.52^ef^	166.5 ± 1.96^efg^	18.20 ± 0.92^ab^	0.60 ± 0.39^a^
	CL	73.00 ± 0.83^a^	321.2 ± 5.71^a^	9.81 ± 1.66^c^	0.42 ± 0.13^a^
	PL	62.04 ± 0.62^cd^	195.9 ± 5.19^d^	17.91 ± 1.48^ab^	0.68 ± 0.18^a^
Mode 2	CU	59.96 ± 1.3^def^	167 ± 2.03^efg^	16.93 ± 0.70^ab^	0.43 ± 0.08^a^
	PU	59.92 ± 0.16^def^	178.2 ± 0.16^e^	15.87 ± 1.83^abc^	0.61 ± 0.28^a^
	CL	60.67 ± 0.59^def^	174.9 ± 1.54^ef^	17.60 ± 1.68^ab^	0.55 ± 0.14^a^
	PL	61.89 ± 0.27^cde^	195.5 ± 1.47^d^	18.24 ± 3.39^ab^	0.56 ± 0.12^a^
Mode 3	CU	63.62 ± 0.38^c^	200.7 ± 1.98^d^	14.40 ± 0.50^abc^	0.51 ± 0.14^a^
	PU	64.48 ± 0.59^c^	226.7 ± 5.10^c^	15.07 ± 2.44^abc^	0.64 ± 0.10^a^
	CL	58.44 ± 0.56^f^	158.5 ± 2.19^g^	19.68 ± 3.24^a^	0.35 ± 0.08^a^
	PL	58.45 ± 0.79^f^	164.1 ± 0.69^fg^	19.71 ± 2.64^a^	0.43 ± 0.08^a^

The values in the table are the mean ± standard deviations. The different lower-case letters in the same column represent significant differences (*p* < 0.05). CU, central upper layer; PU, peripheral upper layer; CL, central lower layer; and PL, peripheral lower layer.

Compared with the rice cooked using mode 1, the cooked rice of mode 3 had a low relaxation time and a small T_23_ peak area, indicating the rice cooked by mode 3 had a more restrictive microenvironment, which agreed with the microstructure of cooked rice.

### Rice texture

To further investigate whether the cooked rice using the three heating modes had different textures, the moisture content, expansion rate, and texture characteristics of rice were analyzed. In general, the expansion rate of cooked rice was positively correlated with moisture content (28), while the hardness of rice was negatively correlated with moisture content (38, 39). As shown in Table 2, modes 1 and 3 displayed varied moisture content, expansion rates, and hardness at different positions in the pot, but rice adhesiveness was similar. In contrast, these characteristics of rice from mode 2 showed no significant differences.

During the cooking process, the moisture content of rice at different positions in the pot was mainly affected by cooking temperature and water absorption time (13, 40). The water absorption rate was positively correlated with the two factors, especially when the cooking temperature was above 60°C (25). Thus, the temperature differences in the rapid heating stage caused differences in the moisture content. In addition, the water absorption time depended on its location in the pot, which indicates the rice closer to the bottom of the pot had longer water absorption time. In mode 1, the higher cooking temperature and longer water absorption time (mode1-CL, Figure 1), resulted in higher moisture content (mode1-CL, 73.00%). Combined with the microstructure of cooked rice, the higher moisture content led to a large cavity structure, which might be the cause of large expansion rate and low hardness. In contrast, lower cooking temperature (mode 1-PU, Figure1) and shorter water absorption time, led to lower moisture content (mode1-PU, 59.21%) and expansion rate and higher hardness, which was consistent with the moisture distribution characteristics and microstructure. Thus, the rice cooked using mode 1 exhibits poor texture uniformity. In mode 3, the moisture content at PU and CU was higher than that at PL and CL because of the higher cooking temperature curve. This indicates cooking temperature differences in the rapid heating stage contributed more than water absorption time to the water absorption differences of rice. In mode 2, the cooking temperature differences compensated for the water absorption time differences between the upper layer (CU and PU) and the lower layer (CL and PL), presenting similar moisture content of rice at the four sampling positions in the pot. Compared to modes 1 and 3, rice cooked at mode 2 presented the best texture uniformity. Above all, an appropriate cooking temperature distribution during the rapid heating stage in the pot should lead to the best texture uniformity.

### Digestion properties of rice

**FIGURE 5:**
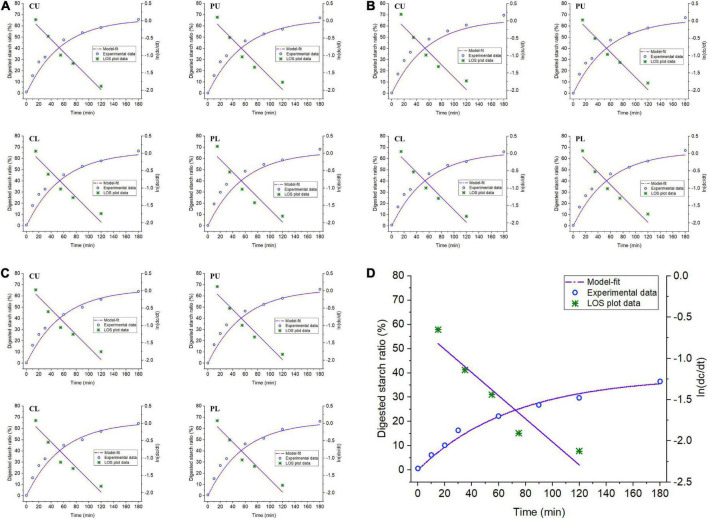
Starch digestion curve and first-order kinetic fitting of raw rice and rice cooked under different heating modes. **(A)** Mode 1. **(B)** Mode 2. **(C)** Mode 3. **(D)** Untreated rice. CU, central upper layer; PU, peripheral upper layer; CL, central lower layer; and PL, peripheral lower layer.

The starch digestion curves and first-order kinetic fittings of rice and raw rice under different heating modes of the rice cooker are shown in Figure 5. Raw rice and rice starch under different heating modes were rapidly digested in the first 30 min of *in vitro* digestion, and then the hydrolysis rate gradually decreased with the extension of digestion time. The starch digestion rate parameters were obtained based on the linear fitting of the LOS diagram (Table 3). Compared with the raw rice, the cooked rice had a significantly reduced resistant starch (RS) content, while the rapidly digestible starch (RDS) and slowly digestible starch (SDS) starch content significantly increased (41). However, there were no significant differences in the content of RS, RDS, and SDS among the rice at different sampling positions under different heating modes. This is consistent with the improved digestibility of rice after cooking. Additionally, no significant difference in equilibrium hydrolysis rate of rice cooked under different heating methods was detected. Schematic diagrams and the SEM data of rice showed rice cooked under different heating modes underwent water absorption and gelatinization, despite slight differences in gelatinization degree, thus forming a loose porous structure. This might explain why the starch hydrolysis rate of the cooked rice samples was significantly improved. The porous structure of rice can enhance the diffusion and accessibility of enzymes in the matrix, contributing to the increase in the digestion rate of rice (42). This may be because the powder samples used in this study further reduced the influence of the internal and external structure of cooked rice on the digestion rate of rice. A similar observation was reported in a previous study (43).

**TABLE 3:** Starch digestion characteristic parameters of raw rice and rice cooked under different heating modes.

Mode	Position	C_∞_ (%)	k × 10^–2^ (min^–1^)	RDS content (%)	SDS content (%)	RS content (%)
Mode 1	CU	66.36 ± 1.22^a^	1.80 ± 0.14^a^	38.93 ± 2.27^a^	44.67 ± 4.09^a^	16.40 ± 3.28^a^
	PU	66.16 ± 1.92^a^	1.75 ± 0.04^a^	40.59 ± 1.49^a^	42.39 ± 2.84^a^	17.02 ± 3.81^a^
	CL	65.32 ± 3.95^a^	1.64 ± 0.07^ab^	39.81 ± 1.62^a^	43.48 ± 1.85^a^	16.72 ± 2.66^a^
	PL	65.17 ± 3.33^a^	1.86 ± 0.03^a^	43.06 ± 3.70^a^	42.02 ± 2.63^a^	14.92 ± 4.16^a^
Mode 2	CU	68.90 ± 4.57^a^	1.78 ± 0.05^a^	42.33 ± 2.92^a^	45.95 ± 3.85^a^	11.72 ± 6.13^a^
	PU	67.19 ± 3.16^a^	1.67 ± 0.12^ab^	38.99 ± 4.61^a^	45.52 ± 3.33^a^	15.48 ± 5.39^a^
	CL	65.38 ± 3.15^a^	1.73 ± 0.08^a^	39.83 ± 3.21^a^	42.53 ± 2.91^a^	17.63 ± 5.19^a^
	PL	66.37 ± 2.55^a^	1.68 ± 0.12^ab^	38.71 ± 2.87^a^	44.93 ± 1.44^a^	16.36 ± 3.70^a^
Mode 3	CU	64.52 ± 3.27^a^	1.62 ± 0.00^ab^	36.99 ± 4.34^a^	45.66 ± 1.20^a^	17.34 ± 5.07^a^
	PU	66.17 ± 3.16^a^	1.81 ± 0.04^a^	38.48 ± 5.48^a^	45.78 ± 0.47^a^	15.73 ± 5.86^a^
	CL	63.70 ± 2.98^a^	1.74 ± 0.09^a^	38.82 ± 4.15^a^	44.29 ± 1.87^a^	16.89 ± 4.68^a^
	PL	66.92 ± 2.96^a^	1.72 ± 0.19^a^	38.21 ± 5.13^a^	47.11 ± 1.87^a^	14.68 ± 5.88^a^
Untreated rice	/	38.66 ± 1.37^b^	1.41 ± 0.03^b^	13.81 ± 2.53^b^	28.54 ± 0.66^b^	57.65 ± 3.01^b^

The values in the table are the mean ± standard deviation and different lower-case letters in the same column represent significant differences (*p* < 0.05). C_∞_, the equilibrium hydrolysis rate of starch; k, the kinetic constant; RDS, rapidly digestible starch; SDS, slowly digestible starch; and RS, resistant starch.

## Conclusion

In this study, the effects of three typical heating sources of rice cookers on the water absorption characteristics, texture, morphological structure, and digestion characteristics of rice at different sampling positions in the pot were investigated. The different cooking modes affected the texture and tissue structure of the entire pot of rice, but there was no significant difference in the digestibility of rice. For mode 2, the temperature consistency in the pot was high, resulting in greater texture, appearance uniformity and similar moisture binding state of rice in the entire pot. For mode 1, the heating rate of the rice at the center position was the fastest across the entire pot, leading to the poorest moisture content, expansion ratio and hardness uniformity of rice samples from different position in the pot. The cooked rice of mode1-CU and mode1-CL were prone to disruption and deformation. Moreover, the microstructure was looser and more porous. For mode 3, the bottom center is the farthest from the heat source, and the temperature was relatively low during the high-temperature maintenance stage. Therefore, the lower layer of rice had a lower moisture content and expansion rate, which resulted in a tighter microstructure, and greater hardness. Compared to modes 1 and 3, rice cooked at mode 2 presented the best texture uniformity. Therefore, this study provides theoretical guidance to improve the texture uniformity of cooked rice in induction heating cooker.

## Data availability statement

The original contributions presented in this study are included in the article/supplementary material, further inquiries can be directed to the corresponding author.

## Author contributions

JK: data curation, investigation, and writing—original draft preparation. JT: project administration, investigation, and methodology. SF: data curation and methodology. YW: formal analysis and validation. SZ: conceptualization, supervision, and writing—review and editing. BZ: conceptualization, supervision, and writing—review and editing. All authors contributed to the article and approved the submitted version.
